# Employment Precariousness and Poor Mental Health: Evidence from Spain on a New Social Determinant of Health

**DOI:** 10.1155/2013/978656

**Published:** 2013-02-03

**Authors:** Alejandra Vives, Marcelo Amable, Montserrat Ferrer, Salvador Moncada, Clara Llorens, Carles Muntaner, Fernando G. Benavides, Joan Benach

**Affiliations:** ^1^Departamento de Salud Pública, Facultad de Medicina, Pontificia Universidad Católica de Chile, 8330073 Santiago, Chile; ^2^Health Inequalities Research Group, Employment Conditions Knowledge Network (GREDS-EMCONET), Department of Political and Social Sciences, Universitat Pompeu Fabra, 08005 Barcelona, Spain; ^3^Center for Research in Occupational Health (CISAL), Department of Experimental and Health Sciences, Universitat Pompeu Fabra, 08003 Barcelona, Spain; ^4^Departamento de Ciencias Ambientales, Universidad Nacional de Avellaneda, B1870BWH Ciudad de Avellaneda, Argentina; ^5^Department of Sociology, Faculty of Political Science and Sociology, Universitat Autònoma de Barcelona (UAB), 08193 Bellaterra, Barcelona, Spain; ^6^Unitat de Recerca en Serveis Sanitaris, Institut Municipal d'Investigació Mèdica, 08003 Barcelona, Spain; ^7^CIBER Epidemiología y Salud Pública (CIBERESP), Spain; ^8^Union Institute of Work Environment and Health (ISTAS), 08003 Barcelona, Spain; ^9^Bloomberg Faculty of Nursing, University of Toronto, Toronto, ON, Canada M5T 1P8

## Abstract

*Background.* Evidence on the health-damaging effects of precarious employment is limited by the use of one-dimensional approaches focused on employment instability. This study assesses the association between precarious employment and poor mental health using the multidimensional Employment Precariousness Scale. *Methods.* Cross-sectional study of 5679 temporary and permanent workers from the population-based Psychosocial Factors Survey was carried out in 2004-2005 in Spain. Poor mental health was defined as SF-36 mental health scores below the 25th percentile of the Spanish reference for each respondent's sex and age. Prevalence proportion ratios (PPRs) of poor mental health across quintiles of employment precariousness (reference: 1st quintile) were calculated with log-binomial regressions, separately for women and men. *Results.* Crude PPRs showed a gradient association with poor mental health and remained generally unchanged after adjustments for age, immigrant status, socioeconomic position, and previous unemployment. Fully adjusted PPRs for the 5th quintile were 2.54 (95% CI: 1.95–3.31) for women and 2.23 (95% CI: 1.86–2.68) for men. *Conclusion.* The study finds a gradient association between employment precariousness and poor mental health, which was somewhat stronger among women, suggesting an interaction with gender-related power asymmetries. Further research is needed to strengthen the epidemiological evidence base and to inform labour market policy-making.

## 1. Introduction

Precarious employment and unemployment, key social determinants of health [1], affect numerous workers in developed and developing countries [2], warranting concern among public health researchers. But, while there is solid evidence of the adverse effects of job loss on health [2], and despite the rapid increase of precarious employment over the past three decades, research on its health effects is limited by the lack of an appropriate measurement instrument to assess precarious employment [3].

During good part of the 20th century, precarious employment in industrialized countries was confined to minority worker subpopulations [4]. Today, it has expanded with the shift undertaken by these countries towards more flexible labour arrangements [2, 5, 6] and the resulting decline of the “standard” employment relationship (full-time, permanent jobs with benefits) that became the norm in the decades following WWII [2].

Precarious employment has been defined in terms of the erosion of the “standard” employment relationship as an employment situation that involves instability, low wages, lack of regulatory protection, and limited worker control over the labour process, or some combination of these. While the erosion of the standard employment relationship affects the workforce as a whole, it is generally women, young workers, less qualified workers, minorities, immigrant workers, and the long-term unemployed who bear the largest share of precarious employment [4, 6, 7].

The extent of precariousness of employment in any given country is contingent on the social, economic, and political processes driving labour market and welfare state policies [8]. A strong welfare state protects workers from financial insecurity during unemployment and other interruptions of their working capacity, leading to the *decommodification* of labour, or the capacity to maintain a livelihood without relying on the market [9]. Taking into account the limitations associated with the gendered character of standard employment relationships and hence, *decommodification*, the notion is useful to understand how welfare state and labour market regulation interact to determine employment conditions [8].

In Spain, the building of the welfare state was late in comparison to other EU countries, being most intensive during the 1980s and 1990s, in the context of a late transition to democracy [10, 11]. The country reached an advanced development of its social security system, but in terms of social expenditure, lagged behind most European countries for the last decade [12]. According to labour market regulation, namely, employment protection legislation (EPL), Spain has been classified as having corporatist conservative labour market institutions [2, 13, 14]. This implies a combination of low union density and high employment protection legislation for both permanent and temporary workers. In fact, formal participation in unions in Spain is low, but collective bargaining coverage is significantly higher (14% and 60%, resp., in 2004) [11]. However, successive labour market reforms have led to a progressive segmentation of the labour force, with a core of permanent employees having stronger protection relative to the more precarious temporary employees, and more recently, to a general weakening of employment protection legislation for both permanent and temporary workers [11, 15]. Characterized, since the mid-1980s, by unemployment rates and shares of temporary employment well above that of other EU-15 countries, Spain's labour market exhibits a high prevalence of precarious employment, even during periods of increased economic activity and low unemployment, affecting not only temporary, but also permanent employees [16]. 

Precarious employment is hypothesized to impact workers' health through several mechanisms: acting as a workplace stressor [17], through social and material deprivation [2], imposing limitations on workers' personal life (such as in their capacity to plan for their future) [17, 18], as well as through hazardous work environments [2], low occupational health and safety standards [5], intermittent unemployment [3], employment strain [18], and sickness presenteeism [19, 20].

Most evidence available today is provided by a significant body of international research focused on the detrimental health effects of job instability. Two main approaches have been used to assess job instability: perceived job insecurity [19, 21] (overall concern about the continued existence of the job in the future) [22], and temporary (atypical, contingent, or nonstandard) employment [5, 20]. This research has demonstrated consistent associations between job instability and various health outcomes, especially regarding poor psychological health [19, 23, 24], as well as workplace injuries and sickness presenteeism in the case of temporary employment [19, 20]. Despite this, and the centrality of job instability to the concept of precarious employment, these research approaches have some important conceptual limitations which have been discussed extensively elsewhere [3, 16, 17].

Briefly, by focusing on job instability, these constitute one-dimensional approaches, providing an incomplete picture of precarious employment [3]. Second, perceptions of job insecurity, a well-accepted feature of precarious employment, may be brought on by contextual—or other—conditions [19] (e.g., sectoral decline, growing shares of nonpermanent employment) above and beyond the extent of precariousness of the current job [25], and as a subjective appraisal of such conditions, may be closer to individual psychology than to the actual conditions and social relations of employment [17, 26]. Third, despite a high degree of overlap between precarious and nonpermanent employment, the latter cannot be unequivocally characterized as health-damaging; there is large heterogeneity within nonpermanent employment arrangements, which gives rise to conflicting research findings [20, 27]. Finally, because permanent employment is usually identified as the ideal standard employment reference, the spread of precariousness into permanent work is neglected, probably leading to underestimation of the association between precarious employment and health. 

Aiming to overcome some of these conceptual and methodological limitations, Amable and colleagues developed and operationalized *employment precariousness *construct [17] and the Employment Precariousness Scale (EPRES) [25]. *Employment precariousness* is a six-dimensional construct encompassing contractual features of precarious employment and workplace social dimensions of employment relationships. The dimensions reflecting contractual aspects of the contract are employment instability (type and duration of the contract), low wages (and possible economic deprivation), limited worker rights and social protection, and individualized contracts (individual-level bargaining over employment conditions). The workplace power relations dimensions are worker vulnerability or defencelessness (to workplace authoritarian, abusive, or threatening treatment), and powerlessness to exercise legal rights [15, 28]. 

Among these dimensions, workplace power relations are a distinctive feature of the employment precariousness construct and have been previously identified as highly relevant for workers' mental health [17, 25]. Both the relaxation of protective regulations and the individualisation of employment relationships (disempowerment) contribute to the exacerbation of workplace power imbalances between management and labour, by providing workers with fewer resources to resist workplace authority and discipline and to exercise workplace rights [3, 29]. Power asymmetries may have nonmaterial links to poor mental health, acting as a workplace stressor [30] and leading to discriminatory workplace practices [17]; and material links, through the unequal distribution of material resources and exposures [2].

Poor mental health is proposed as the most likely health outcome of *employment precariousness* [17]. Quantitative research suggests job insecurity and temporary employment are most consistently and significantly associated with mental ill health [19, 21]. Qualitative research conducted in Canada found that most workers in nonpermanent employment reported work-related stress and poor mental health, whereas physical health appeared subject to harm in the longer-run [18]. In Spain, developers of the construct conducted qualitative research among Spanish [17] and immigrant workers [7], describing mental ill health to be at the core of interviewees' complaints regarding employment precariousness. 

The purpose of this study is to contribute with quantitative evidence regarding the association between *employment precariousness*, as measured with the EPRES, and poor mental health among waged and salaried workers in Spain.

## 2. Methods

### 2.1. Survey Design and Study Population

Data come from the Union Institute of Work, Environment and Health (ISTAS) Psychosocial Work Environment Survey (PWES), a cross-sectional population-based survey carried out between October 2004 and July 2005 on a representative sample of the wage-earning population living in Spain (*n* = 7650), where the EPRES was included [31]. While the survey was conducted, nonpermanent employment in Spain accounted for 33% of waged work, a stable proportion since 1990, and unemployment rates fell from 10.6% to 8.4% [32].

Sample selection followed a multistage, stratified, random-route sampling procedure. Questionnaires were administered at the respondents' homes by trained interviewers. Subjects were eligible if they were aged 16 to 65 and had worked in a paid employment job for at least one hour during the week preceding the survey (including employed subjects absent from their job). Nonrespondents were substituted on the field, following the same sampling procedures and inclusion criteria. Fieldwork was conducted during autumn, winter, and spring to account for seasonal variations in economic activity, while avoiding the summer season due to difficulties in recruitment during summer holidays. The response rate was 60%. The survey was voluntary and confidential, and the dataset was completely deidentified before analysis. Prior to its initiation, the PWES protocol was reviewed and approved by ISTAS institutional review board (IRB).

Given that EPRES was devised for waged workers with a contract [25], we restricted our analyses to permanent and temporary workers with a contract, including temporary agency workers. We excluded self-employed workers, workers without a contract, graduate students, and workers with unknown employment status (*n* = 684). Respondents of noneligible ages were also excluded (*n* = 19). To provide for an induction period and to avoid confounding by the mental-health effects of previous unemployment and recent reemployment, which appear to be strongest during the first 6 months [33], the sample was further restricted to employees with tenures of six months or longer, excluding subjects with shorter (*n* = 845) or unknown (*n* = 37) tenure. Finally, all subjects with nonresponse to any of the study variables were excluded (*n* = 388). Differences in the distribution of study variables between respondents with complete data and those with missing data were not statistically significant (Chi-square tests, *P* values ≥0.05; results not reported but available upon request). The final sample size was 5679.

### 2.2. Study Variables

#### 2.2.1. Employment Precariousness

The EPRES is a structured questionnaire, validated among waged workers with either a temporary or permanent contract [25]. It comprises 26 items grouped into six subscales: “instability” (contract duration), “disempowerment” (individual-level bargaining over, e.g., wages, working hours), “low wages” (monthly wage/salary, capacity to cover regular or unexpected expenses), “rights” (entitlement to workplace rights such as sick leave, weekly rest, vacations), “vulnerability” (defencelessness to, e.g., unfair, violent, authoritarian treatment), and “capacity to exercise rights” (e.g., maternity/paternity leave, vacations). Subscale scores were computed as simple averages, transformed into a 0–4 scale, and averaged into a summary score ranging from 0 to 4 [25], which was grouped into quintiles. Following recommendations derived from a previous study on the psychometric properties of the EPRES, nonresponse to one item in the “wages” dimension (monthly wage/salary) was allowed for [25]. 

#### 2.2.2. Mental Health

General mental health was assessed with the Spanish version of the 5-item Mental Health (MH) scale of the Short Form-36 Health Survey (SF-36) [34], which taps feelings of nervousness, anxiety, depression, and psychological wellbeing during the preceding four weeks [35]. The MH score is calculated as the sum of the 5 items, transformed into a 0–100 score. Low scores indicate psychological distress, while high scores indicate psychological wellbeing.

Because no formal cut-off scores have been established, general population-based reference norms have been the interpretation strategy most recommended for the SF-36 questionnaires. Normative data facilitates score interpretation by comparing the study sample to the normative population [36]. Applying Spanish reference norms obtained in 1996 from a representative sample of the general population [34], we defined poor mental health status as a score below the 25th percentile (the lowest quintile) of the Spanish reference for the individual's sex and age.

#### 2.2.3. Sociodemographic Variables

Demographic variables used were sex, age (for descriptive purposes, age was grouped into five categories corresponding to the SF-36 reference groups: 16–24, 25–34, 35–44, 45–54, and 55–65 years of age), immigrant status (yes/no, according to the responder's reported country of origin), unemployment during the year preceding the survey (yes/no), and socioeconomic position.

Two socioeconomic position (SEP) indicators were used: level of educational attainment and occupational class. Highest completed level of education was grouped into four strata: primary or less, secondary, trade school, and university. Occupational class was obtained following the Spanish Epidemiological Society proposal for a social class measure [37] and grouped into three strata: higher and lower managerial and professionals (SC I + II), administrative personnel and supervisors (SC III), and skilled, semiskilled, and unskilled manual occupations (SC IV + V).

### 2.3. Analyses

Study variables were described as sample counts and percentages. Mean mental health scores were described for men and women in each age group and tested for trends with weighted ANOVA tests. Crude associations between the study variables and poor mental health were described and tested for significance using Pearson *X* ^2^ (categorical variables) tests or weighted Anova tests for linear trends (ordinal variables).

We used multivariate log-binomial regressions to estimate adjusted prevalence proportion ratios (PPRs) of poor mental health and their 95% confidence intervals. Prevalence proportion ratios [38], and not prevalence odds ratios, were calculated because of the cross-sectional nature of the study and the high-prevalence outcome being studied. The model output is the PPR of poor mental health in quintiles 2, 3, 4, and 5 of employment precariousness as compared to quintile 1, the lowest precariousness level (reference group).

Three models are presented: model 1, adjusted for age (continuous); model 2, adjusted for age, immigrant status, educational attainment, and occupational class; and model 3, further adjusted for previous unemployment. Adjustments were aimed at controlling for the potential impact of social position on health through pathways unrelated to employment precariousness, and for potential confounding by previous unemployment [20], which is associated with poor mental health [2] and predictive of precarious reemployment [6]. Education and occupational class were included simultaneously in the models to capture life-course information on SEP [39].

In additional analyses we tested for PPR trends with the Wald statistic by introducing a continuous variable representing the ordinal categories (quintiles) of precariousness into the models. All analyses were stratified by sex, given different role configurations of women and men [40], and that employment precariousness has been hypothesised to have a greater impact on women's health [41]. Analyses were performed using the SPSS 15.0 programme.

## 3. Results

The study sample included 2709 women and 2970 men. For both women and men, the majority of respondents were between 25 and 44 years old, Spanish, had achieved secondary education or higher, were manual workers, and had not been unemployed during the previous year (Table 1). Compared to men, women were younger, more frequently university graduates, less frequently in manual occupations, reported more unemployment and higher levels of employment precariousness. 

**Figure 1:**
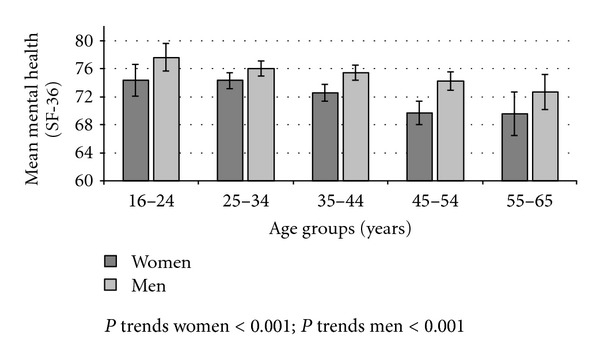
Mean mental health scores (95% CI) according to age groups. Waged and salaried women and men, Spain 2004-05.

Mean mental health scores were higher (better) among men than women across all age groups and decreased with age for both (*P* for trends <0.001) (Figure 1). Poor mental health was reported by 29.4% of men and 22.5% of women, showing a tendency to decrease with age among women and to increase among men. The highest prevalence of poor mental health was reported by women aged 25–34 and by men aged 45–54. The prevalence of poor mental health was significantly higher among workers with lower educational attainment, manual workers (SC IV + V), those who had been previously unemployed, immigrant workers (among women only), and increased as employment precariousness increased, being twice as high in the 5th as in the 1st quintile among men, and 2.8 times as high among women (Table 2).

**Figure 2:**
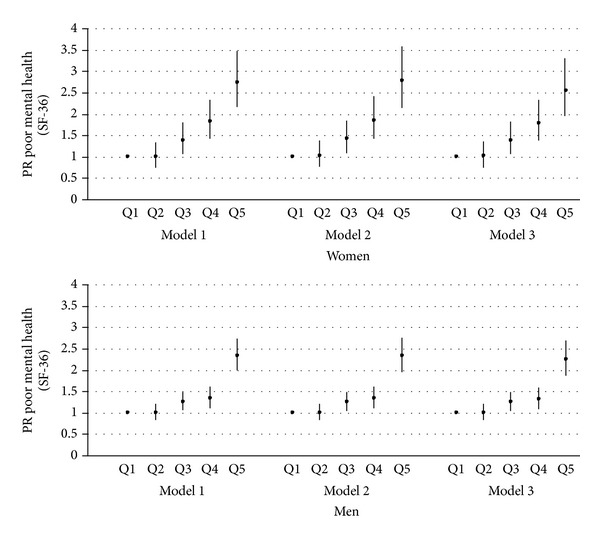
Prevalence proportion ratios (95% CI) of poor mental health according to quintiles of employment precariousness. Waged and salaried women and men, Spain 2004-05. Model 1: adjusted for age (continuous). Model 2: adjusted for age, immigrant status (yes/no), educational attainment (primary or less; secondary; trade school; university), and occupational social class (SC I + II; SC III; SC IV + V). Model 3: model 2 + unemployment the previous year (yes/no).

In the adjusted models, crude associations remained generally unchanged despite the adjustments performed. Fully adjusted (model 3) PPRs in women were 1.01 (95% CI: 0.75–1.36) for the 2nd quintile; 1.39 (95% CI: 1.05–1.82) for the 3rd quintile; 1.78 (95% CI: 1.37–2.32) for the 4th quintile; and 2.54 (95% CI: 1.95–3.31) for the 5th quintile. In men these were 1.00 (95% CI: 0.83–1.21) for the 2nd quintile; 1.24 (95% CI: 1.03–1.49) for the 3rd quintile; 1.31 (95% CI: 1.08–1.59) for the 4th quintile; and 2.23 (95% CI: 1.86–2.68) for the 5th quintile. Trends were significant for both women and men in the three models (*P* < 0.001) (Figure 2). We tested for the significance of the observed difference between women and men in a single, fully adjusted, model for both women and men, including the interaction between quintiles of employment precariousness and gender. We found significant (*P* < 0.10) [42, 43] interaction effects for the 4th quintile of precariousness (*P* = 0.02), and close to significant for the 5th quintile (*P* = 0.11), in which the association confirmed to be stronger for women than men (data not shown).

To ensure our findings were not dependent on the cut-off scores we used to identify subjects in poor mental health (based on Spanish reference values) [34], we repeated our analyses using sample-based, gender-specific 25th percentiles of mental health as cut-off scores. The gradient and magnitude of associations between employment precariousness and poor mental health were highly similar to our study results (data not shown). The largest change in the fully adjusted model was for the 5th quintile in women (PPR: 2.38; 95% CI: 1.91–2.96) and in men (PPR: 2.40; 95% CI: 1.96–2.93).

Monthly wages or salary, included in the “wages” dimension, make up an important part of income, another indicator of socioeconomic position. To ensure our findings were not explained by income alone, we repeated the multivariate analyses excluding the “wages” dimension from the EPRES score and including “monthly wage/salary” (11 income brackets) as a covariate (data not shown) in the models. In comparison to our original results, observed associations exhibited minor changes: fully adjusted PPRs for the 5th quintile were 2.23 (95% CI: 1.77–2.81) in women and 2.18 (95% CI: 1.83–2.59) in men.

## 4. Discussion

This study is the first population-based study to explore the association between precarious employment and mental health by means of the multidimensional Employment Precariousness Scale. The main study findings were that employment precariousness is associated with poor mental health, even after controlling for potential confounders, that the association increased along a gradient of employment precariousness in a dose-response pattern, and that the association was slightly stronger among women than men.

The general hypothesis that employment precariousness is associated with poor mental health was supported by our results: among workers in the 5th quintile of employment precariousness, the prevalence of poor mental health more than doubled that of workers in the 1st quintile. These results reinforce preexisting qualitative research findings which describe how the various dimensions of employment precariousness contribute to the deterioration of workers' mental health [7, 17].

The observation of a gradient association between employment precariousness and poor mental health supports the notion that precarious employment is not a dichotomous phenomenon [3, 20] and cannot be well captured by crude research categories such as standard/nonstandard employment [4]. In fact, standard employment is an ideal type against which to compare real-life employment relations, but not even permanent employment conforms to this ideal. 

The hypothesis that employment precariousness has a stronger impact on women's than men's mental health was supported by the data: the slope of the gradient was steeper in women, and overall associations were stronger in them. Employment-related workplace power asymmetries affect both women and men, but their interaction with gender-related power asymmetries within or without the workplace might explain a stronger association among women [41]. Within the workplace, gender may structure the access to organizational power and informal sources of power [44]. Outside the workplace, employment precariousness may be interacting with the gendered distribution of the domestic workload [40], in particular in Spain where working women, and especially manual working class women, continue to perform most domestic chores and have fewer resources to face the conflicting demands of paid and unpaid work, resulting in a greater work overload and stress [45–47]. An alternative explanation could be that women are more vulnerable to stressful life conditions; previous unemployment research, however, suggests that such differences are dependent on the configuration of roles, not on intrinsic gender vulnerabilities [40].

In addition to our main study results, the sample distribution of poor mental health deserves some commentary. While mental health scores behaved as expected, that is, were higher (better) in men than women, and decreased with age in both, when compared to the Spanish reference norm [34] male respondents had a higher prevalence of poor mental health than female respondents. Further, women's prevalence of poor mental health, but not men's, tended to decrease with age. A French study using national thresholds on a mental health scale applied to almost 12 000 workers found that when used as a continuous score, women's mental health was worse than men's; when thresholds were used, no such differences were observed [48]. While these findings may be partially accounted for by demographic and socioeconomic differences between ours and the normative sample, they are also suggesting a stronger healthy worker effect among women than among men, especially at older ages. This is consistent with previous multinational research findings [49] which suggest that continued health-related selection out of the workforce is stronger in women than men. The higher prevalence of poor mental health among employed women around their thirties may also be related to the difficulties involved in balancing work and family responsibilities [50]. Future research is necessary to better understand the differential health-related selection of men and women into and out of the workforce. 

Our study has the strength of being a population-based study [20] performed on a large, representative sample of the Spanish workforce and of using a multidimensional, theory-based, validated, measure of precarious employment, a well-validated measure of mental health, and relevant social stratification variables as well as a measure of previous unemployment to control for potential confounding. Because employment precariousness was measured among both temporary and permanent workers, contract type, although contributing to the overall score, did not determine the classification of study subjects into the exposure categories, thus overcoming limitations of previous research using employment status as an indicator of precariousness. In addition, this has methodological implications for research on mental health and unemployment: studies that do not control for precariousness might be vulnerable to misclassification error (i.e., the nonexposed group may be exposed to the risks of precarious employment). 

However, the study has the limitations of cross-sectional data for drawing causal inferences: observed associations could be explained by reverse causation due to health selection. Previous qualitative research has proven informative in this regard, supporting the causal link between precarious employment and poor mental health [7, 17, 18]. Prospective quantitative research has provided evidence both in favour of causation [51, 52] and of health selection [53, 54], although effects are typically stronger for the former [55]. 

The impact of health selection appears to be twofold. On the one hand, a better health status favours selection into permanent employment. On the other, nonpermanent workers undergo repeated processes of health selection each time they seek employment; and, once employed, less healthy temporary workers are more likely to lose their jobs than equally less healthy permanent workers [56]. Thus, the healthy worker survivor effect (out-selection of less healthy workers) will operate more strongly among temporary employees, while wearing off of selection will be more pronounced among permanent employees [20]. To the extent that there is an overlap between nonpermanent and precarious employment, health-related selection into permanent employment may be leading to an overestimation of the association between employment precariousness and health, whereas the healthy worker survivor effect and wearing off of selection may be leading to its underestimation [56]. 

Another limitation of our study is that both the measures of exposure and outcome rely on self-reports. Self-reports of mental health can be useful and valid measures of psychological health and predictors of psychological morbidity [57] but due to self-report bias, the association between employment precariousness and self-reported poor mental health may be overestimated [29]. However, there is a weak correlation between employment precariousness and self-reports regarding the psychosocial work environment [25], suggesting that negative reporting might not be affecting the assessment of employment precariousness.

Regarding the study sample, the restriction to employees with a contract may limit the generalizability of these findings to other subgroups of workers such informal workers or workers without a contract and the self-employed, especially the so-called bogus self-employed. Similarly, with the purpose of providing for an induction period, of limiting the influence of previous unemployment on current mental health, as well as the influence of reemployment on current mental health [33], short-tenured (≤6 months) workers were excluded from this study. While this excludes very precarious workers, results are more informative on the association between exposure to current employment precariousness and mental health. Additional analyses were performed, including workers tenured 2 to 6 months, and results remained largely the same (data not shown). Finally, while the study sample resembles the Spanish labour force as measured by the quarterly labour force survey, there are some differences of note: a higher proportion of women in the study sample, although this will not affect external validity because analyses were performed separately for women and men; a lower proportion of temporary workers, and absence of agricultural workers and household service workers, which are among the most precarious groups [16]. This calls for caution in the generalization of the study results to these groups of workers.

This study contributes with quantitative evidence to qualitative research on employment precariousness and health, and to previous epidemiological research on employment conditions and health by expanding the focus beyond the instability dimension of flexible work contracts. The observed gradient association between employment precariousness and poor-mental health highlights the relevance of employment conditions for worker wellbeing. 

However, further research is needed to strengthen the epidemiological evidence base and to inform labour market policy-making. Among the future developments, research should explore the pathways linking employment precariousness and health and explore other, possibly longer-term, health outcomes, address the gender-related issues raised by this study, explore differences across other groups such as manual and nonmanual workers, and further this area of inquiry into other national contexts, other employment arrangements, such as informal and dependent self-employed workers, and other groups of vulnerable workers.

## Figures and Tables

**Table 1:** Sample characteristics. Waged and salaried women and men, Spain 2004-05.

	Women	Men
Total	2709 (47.7%)	2970 (52.3%)
Age group		
16–24 years	273 (10.1%)	253 (8.5%)
25–34 years	963 (35.5%)	848 (28.6%)
35–44 years	875 (32.3%)	973 (32.8%)
45–54 years	463 (17.1%)	667 (22.5%)
55–65 years	135 (5.0%)	229 (7.7%)
Immigrant status		
Spanish	2555 (94.3%)	2762 (93.0%)
Immigrant	154 (5.7%)	208 (7.0%)
Educational attainment		
Primary or less	776 (28.6%)	1109 (37.3%)
Secondary	780 (28.8%)	850 (28.6%)
Trade school	433 (16.0%)	452 (15.2%)
University	720 (26.6%)	559 (18.8%)
Occupational class^a^		
SC I + II	493 (18.2%)	487 (16.4%)
SC III	722 (26.7%)	581 (19.6%)
SC IV + V	1494 (55.1%)	1902 (64.0%)
Unemployment preceding year		
No	2468 (91.1%)	2793 (94.0%)
Yes	241 (8.9%)	177 (6.0%)
Quintiles of employment precariousness		
0.00–0.61	524 (19.3%)	745 (25.1%)
0.62–0.85	551 (20.3%)	686 (23.1%)
0.86–1.12	568 (21.0%)	635 (21.4%)
1.13–1.55	578 (21.3%)	523 (17.6%)
1.56–4.0	488 (18.0%)	381 (12.8%)

^a^SC I + II: higher and lower managerial and professional; SC III: administrative personnel and supervisors; SC IV + V: skilled, semiskilled, and unskilled manual.

**Table 2:** Prevalence of poor mental health^a^ (percentage and 95% CI) according to age, immigrant status, educational attainment, occupational social class, and unemployment the preceding year. Waged and salaried women and men, Spain 2004-05.

	Women		Men	
	%	(95% CI)	%	(95% CI)
All	22.5	(20.9–24.1)	29.4	(27.8–31.0)
*P* value				*0.000 *
Age group				
16–24 years	19.8	(15.0–24.5)	26.1	(20.6–31.5)
25–34 years	26.8	(24.0–29.6)	27.2	(24.2–30.2)
35–44 years	21.1	(18.4–23.9)	29.8	(26.9–32.7)
45–54 years	19.9	(16.2–23.5)	32.5	(29.0–36.1)
55–65 years	15.6	(9.4–21.7)	30.1	(24.1–36.1)
*p* linear trend		*0.010 *		*0.026 *
Immigrant status				
Spanish	21.9	(21.9–41.4)	29.1	(27.4–30.8)
Immigrant	33.1	(33.1–47.2)	32.7	(26.3–39.1)
*P* value		*0.001 *		*0.279 *
Educational attainment				
Primary or less	25.1	(22.1–28.2)	32.7	(30.0–35.5)
Secondary	22.6	(19.6–25.5)	30.1	(27.0–33.2)
Trade school	18.7	(15.0–22.4)	21.7	(17.9–25.5)
University	21.9	(18.9–25.0)	27.9	(24.2–31.6)
*p* linear trend		*0.008 *		*0.029 *
Occupational social class				
SC I + II	20.1	(16.5–23.6)	27.7	(23.7–31.7)
SC III	19.7	(16.8–22.6)	25.1	(21.6–28.7)
SC IV + V	24.7	(22.5–26.9)	31.1	(29.0–33.2)
*p* linear trend		*0.010 *		*0.014 *
Unemployment preceding year				
No	20.8	(19.2–22.4)	28.3	(26.6–30.0)
Yes	39.8	(33.6–46.1)	46.3	(38.9–53.7)
*P* value		*0.000 *		*0.000 *
Quintiles employment precariousness				
0.00–0.61	14.3	(11.3–17.3)	23.9	(20.8–27.0)
0.62–0.85	14.3	(11.4–17.3)	23.3	(20.2–26.5)
0.86–1.12	19.7	(16.4–23.0)	29.0	(25.4–32.5)
1.13–1.55	26.1	(22.5–29.7)	30.0	(26.1–34.0)
1.56–4.0	39.5	(35.2–43.9)	50.9	(45.9–56.0)
*p* linear trend		*0.000 *		*0.000 *

^a^Poor mental health was defined according to the Spanish reference norm as a score below the 25th percentile for the individual's sex and age (18–24; 25–34; 35–44; 45–54; and 55–64 years). Cut-off scores for women were 60, 63.2, 60, 56, and 52, respectively. Cut-off scores for men were 68, 68, 68, 68, and 64, respectively. Subjects aged 16 or 17 (*n* = 23) were assigned the reference value of the 18–24 age group; respondents aged 65 (*n* = 10) were assigned the reference value of the 55–64 age group.

CI: confidence interval.
